# Estimating QALYs in adults with cerebral palsy: mapping the San Martin scale to the EQ-5D-5L for economic evaluation

**DOI:** 10.1007/s10198-025-01831-1

**Published:** 2025-09-24

**Authors:** Diana Marcela Nova Díaz, Aritz Adin, Eduardo Sánchez-Iriso

**Affiliations:** 1 Department of Economics, Public University of Navarra, Pamplona, Spain; 2 Department of Statistics, Computer Science and Mathematics, Public University of Navarra, Pamplona, Spain

**Keywords:** Mapping, Cerebral palsy, EQ-5D-5L, St. MQoL-S, Utilities, Economic evaluation, I10, I31, J14, C21

## Abstract

**Background:**

Responses on health-related quality of life measured by disease-specific instruments can be mapped onto the EQ-5D to estimate utility values for economic evaluation. San Martin´s Quality of Life Scale (St. MQoL-S) is a preferred measure to obtain health outcomes in adults with cerebral palsy. Nevertheless, it lacks a preference-based health utility score for estimating quality-adjusted life years (QALYs).

**Objective:**

To develop algorithms for mapping from the St. MQoL-S to allow future prediction of the EQ-5D-5L, in adults with cerebral palsy, when utility data have not been collected.

**Methods:**

Direct mapping models were developed using ordinary least squares, a generalized linear model, and Tobit regression analysis to estimate EQ-5D-5L utilities, with St. MQoL-S total and domain scores as explanatory variables, in a cross-sectional study of adults with cerebral palsy in Spain. Goodness-of-fit was assessed using mean absolute error (MAE) and root mean square error (RMSE). Repeated k-fold cross-validation was employed to select the optimal mapping model demonstrating superior predictive performance.

**Results:**

The best-performing model for predicting EQ-5D-5L utilities, includes the St. MQoL-S total scores, age, gender, and types of cerebral palsy as explanatory variables in a stepwise ordinary least squares regression, making it the most robust model for use as a mapping algorithm with external data.

**Conclusion:**

This is the first study to present mapping algorithms between the St. MQoL-S and EQ-5D-5L. The mapping functions preferred in this study seem adequate for estimating the utilities of the EQ-5D-5L for economic evaluation and to obtain QALYs in adults with cerebral palsy.

**Supplementary Information:**

The online version contains supplementary material available at 10.1007/s10198-025-01831-1.

## Background

The constant need to improve the quality of healthcare in the Spanish National Health System (NHS) relies on the ability to assess the quality of both existing and new services over time. With the recent emphasis in the Spanish NHS on value-based contracting, it is necessary to monitor and measure outcomes [1, 2]. Quality Adjusted Life Years (QALYs) are composite measures of life duration and quality of life, and they provide a way to measure the impact of healthcare interventions on health-related quality of life (HRQoL). Cost per QALY is commonly used to assess the cost-effectiveness of interventions and to guide resource allocation. The use of outcome measures internationally [3] as well as in Spain has increased in the last decade [4].

Cerebral palsy (CP) is a neurological disease characterized by permanent movement and/or postural impairments resulting from non-progressive brain damage occurring during development. It is frequently accompanied by comorbidities such as epilepsy, intellectual disabilities, and sensory limitations [5]. Its prevalence in Spain and Europe is approximately 2–3 cases per 1,000 live births, with life expectancy ranging from 30 to 70 years [5, 6]. CP has no cure and significantly impacts HRQoL from early childhood into adulthood.

The economic burden of CP is considerable due to the need for continuous, specialized care [7] such as rehabilitation therapies and support for daily functioning which places a sustained demand on families and health systems [8, 9]. This burden has been moderately documented in international literature, particularly in high-income countries like Australia, Canada, and China, where cost-of-illness (COI) studies estimate annual societal costs per patient between €40,000 and €95,000, depending on severity and care model [10–12]. These costs include loss of caregiver productivity, out-of-pocket expenses for therapies and assistive devices, transportation, and specialized education many of which are only partially covered by public healthcare systems. In Europe, however, COI studies remain limited. One Dutch study from 2010 estimated annual CP-related costs of up to €40,265 per patient [13]. In Spain, however, evidence is similarly scarce, and the absence of a national CP registry complicates reliable cost estimation. To our knowledge, only one recent Spanish study has estimated that the average annual societal cost of CP can reach €102,135 per patient, with families absorbing almost 74% of this burden due to unpaid care and unreimbursed expenses [14]. The lack of robust country-specific data in many settings impedes the development of equitable and cost-effective care strategies tailored to the needs of individuals with CP.

Given the burden and economic impact of CP, it is crucial to improve our knowledge base to guide treatment, prevention, and the potential need for evidence to support health technology assessment and reimbursement decisions. Currently, there is limited literature on health benefits based on the preferences of adult patients with CP [15], which is necessary to measure health effects, through the calculation of QALYs for cost-effectiveness analyses. These types of economic evaluations usually take a social perspective, which means that all costs and effects are included in the analysis regardless of who bears them [16].

The San Martin Quality of Life Scale (St. MQoL-S) is a disease-specific instrument widely used in Spain to assess wellbeing and HRQoL in adults with CP [17, 18]. It is grounded in Schalock and Verdugo’s eight-dimensional quality of life model and has demonstrated strong validity and reliability in this population [19, 20]. Originally designed for use in clinical settings, the St. MQoL-S supports evidence-based practice by enabling the assessment of personal outcomes, guiding person-centered interventions, and informing service evaluation. It is also internationally validated and applied in institutions that care for people with significant disabilities, such as CP [21, 22].

The scale functions both as a direct and proxy measure of HRQoL and is frequently used to evaluate the effectiveness of treatments and interventions [18]. To our knowledge, it is the only HRQoL instrument specifically developed for use in adults with CP, which underscores its relevance and uniqueness [20, 23]. Its widespread use in clinical practice facilitates familiarity among care providers and enhances its applicability. Additionally, it has been shown to be more sensitive than generic instruments in detecting changes in the health status of individuals with CP [20, 22]. However, a key limitation of the St. MQoL-S is that it does not generate preference-based utility values and therefore cannot be directly used to calculate QALYs.

QALYs are generated using preference-based health-related quality of life instruments. The EQ-5D-5L is the most commonly used instrument for this purpose and is recommended by the National Institute for Health and Care Excellence (NICE) and the Spanish NHS [4, 24]. It is a generic, standardized instrument widely used around the world that facilitates comparisons of health technologies across different diseases [16, 25, 26]. It has also been validated as a concise, simple, and effective instrument for measuring HRQoL in people with CP [13, 27]. However, most studies evaluating quality of life in CP populations tend to favour disease-specific quality-of-life instruments [28] or clinical measures of performance status versus generic instruments such as the EQ-5D-5L [17, 29].

Despite growing use of the EQ-5D in economic evaluations, direct utility data for adults with CP are scarce. When EQ-5D-5L data are not available, the NICE allows utilities to be estimated by mapping [3] from other HRQoL instruments [24, 30]. Mapping techniques use either direct (predicting utility scores) or indirect (predicting individual domain scores) approaches. Direct mapping is often preferred when sample sizes are small or when detailed domain-level responses are unavailable. Both approaches have been applied in other neurological populations, but few applications exist for CP [27, 31, 32].

The appropriateness of existing generic HRQoL measures for application in adults with CP, has received limited research attention [15, 33]. In addition, a mapping algorithm specific to adults with CP using a generic instrument for this population, has not yet been developed. Given that some relevant items and/or domains overlap between the St. MQoL-S and the EQ-5D-5L (see Table S1 in the supplementary material), it is plausible that there is a significant relationship between these two instruments. Consequently, we considered developing a mapping algorithm that would allow the calculation of QALYs when investigators wish to explore the cost-effectiveness of an intervention.

This study aimed to develop a mapping algorithm from the St. MQoL-S to allow future prediction of the usefulness of the EQ-5D-5L, in adult patient populations with CP, when utility data have not been collected.

## Methods

The most recent ‘best practices’ for mapping utilities from non-preference-based measures were followed throughout the study and reporting [23]. This study was conducted primarily in accordance with the"Mapping onto Preference-based Measures Reporting Standards"(MAPS) checklist [34]. Additionally, it was complemented by the NICE EQ-5D mapping guide for use in health technology assessment [30], and by the ISPOR Task Force report,"Mapping to Estimate Health-State Utility from Non–Preference-Based Outcome Measures: Good Practices for Outcomes Research [35]".

### Data

The data were obtained from the Adult Cerebral Palsy Study in Navarra (EPCANA), a cross-sectional study to measure HRQoL, clinical outcomes and calculate QALYs in adults with CP. The study was conducted at the Spanish Association for the Care of People with CP (Aspace), as Aspace is a specialized institution in adult cerebral palsy care. In Spain, the public health system does not facilitate continuity of care for this age group due to the lack of a structured transition between pediatric and adult services. This limitation hindered the possibility of obtaining relevant data directly from the public system, making Aspace the primary accessible and reliable source for this research.

### Participants

A total of 110 adults with CP registered in Aspace were initially considered for inclusion in the study. However, only participants with sufficient cognitive ability to independently complete the two quality of life instruments, the St. MQoL-S and the EQ-5D-5L, were included. Because 38 individuals had severe cognitive impairment, the final sample consisted of 72 participants. Cross-sectional data from these participants were collected face-to-face between January and July 2023 by Aspace's clinical and research teams. Participants in the study were part of a specialized Aspace patient-centred care program. This program includes recreational and educational activities both within the institution (residential homes, school and day centers) and outside. In addition, to sessions of therapies such as physiotherapy, occupational therapy, compensatory education and speech therapy, all of which are individually directed and adapted to the needs of each patient. Furthermore, adults with CP participated in Aspace’s occupational center program, which aims to promote social integration through employment. The patients provided demographic and clinical information and completed a series of outcome measures. The data collected, which included complete responses from the St. MQoL-S and EQ-5D-5L instruments, were crucial to develop and identify the most effective mapping algorithm.

The study incorporated a functional dependency classification that distinguishes between three levels: Level I, representing the least dependency, and Level III, reflecting the greatest. This classification was gathered using the San Martín Scale, an instrument employed for participant assessment. Additionally, a classification based on neurological findings was used to categorize the types of CP into spastic, dyskinetic, ataxic, and unclassified.

The Adult Cerebral Palsy Study in Navarra (EPCANA) received ethical approval from the Aspace committee (registration no. 026/23).

### Measures

The EQ-5D-5L is a generic preference-based measure. It is an objective measure by which patients describe their health status [26]. It assesses health-related quality of life in 5 dimensions (mobility, self-care, usual activities, pain/discomfort and anxiety/depression). Respondents can indicate one of five levels of severity: no problems (level 1), slight problems (level 2), moderate problems (level 3), severe problems (level 4), unable to/extreme problems (level 5) in the corresponding health dimension [36, 37]. This scale generates a single preference-based utility index score with any combination of responses, anchored at 0 to represent death and 1 to represent full health. This index is obtained by transforming the dimension scores with country-specific tariffs. Utility values used in this study were computed using the Spanish EQ-5D-5L tariff [38].

**Fig. 1:**
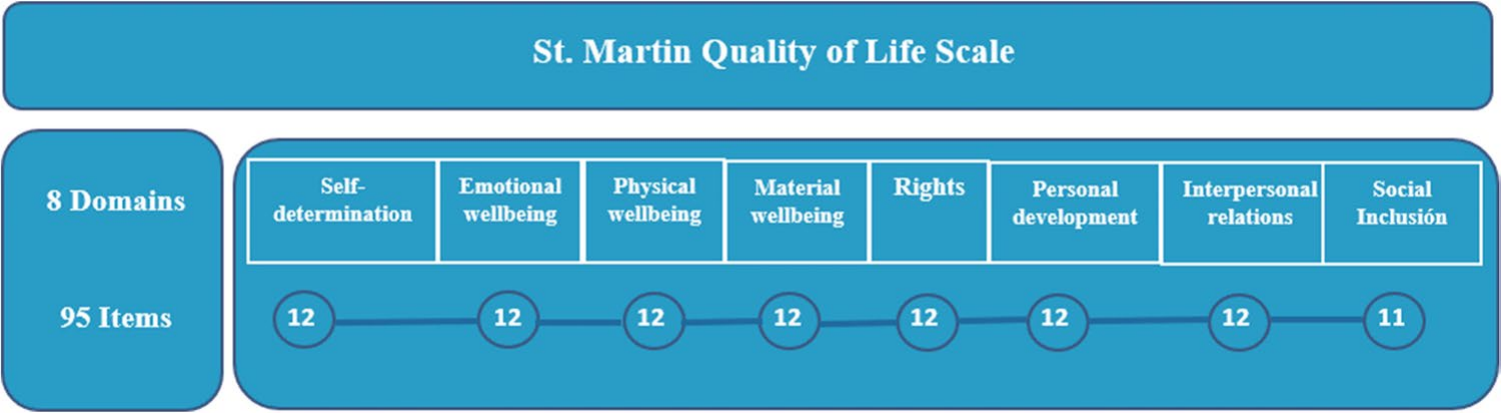
Structure of the San Martin Scale, showing the breakdown into eight domains and ninety-five items. The items are related in each dimension. All dimensions have 12 items, except for the social inclusion domain, which has 11 items. To see the full scale, (see supplementary material, at the bottom of Table S1). Own elaboration, based on the Scale Manual [21]

The St. MQoL-S is a widely used non-preference-based measure of health outcomes in adults with CP. This evaluates a comprehensive, functional, and specific approach to quality of life [18]. It consists of a validated 95-item questionnaire specific to adults with significant disabilities, including CP. The responder is asked to answer the questions with a ‘never’, ‘sometimes’, ‘often’ or ‘always’. Responses to St. MQoL-S are often used to derive a single HRQoL index value and this value is reported in clinical studies. The St. MQoL-S measures HRQoL over a range of values from 52 to 132 points in its quality of life index. A higher score represents higher HRQoL in the *St. MQoL-S index or, in other words, St. MQoL-S total score*. This scale measures HRQoL in 8 dimensions (Fig. 1).

It is an internationally validated instrument, widely used in institutions dedicated to the specialised care of adults with CP [39]. Several studies have attested to its sensitivity, and that it is suitable for direct and proxy assessment in disabilities such as CP [22, 40, 41]. The St. MQoL-S has not been previously mapped to our knowledge.

### Statistical analysis

#### Mapping from St. MQoL-S to the EQ-5D-5L

To develop the mapping functions between the St. MQoL-S and the EQ-5D-5L, we used only direct mapping. An indirect or response mapping approach was not select because it requires a large sample size with an adequate number of responses for each response level, which was not available in this study [30, 31, 42].

Before undertaking the mapping, we assessed whether to use all dimensions of the St. MQoL-S or only selected ones. We first calculated Spearman’s correlation coefficients between each St. MQoL-S domain and the EQ-5D-5L utility index, considering dimensions with correlations below 0.4 as weakly associated. These results are presented in Supplementary Table S2. We then applied *ANOVA F-tests and stepwise regression* using the Akaike Information Criterion (AIC) to evaluate alternative model specifications and identify the most appropriate set of predictors.

#### Variable selection

All available sociodemographic and clinical variables were considered as potential covariates. These included age and sex (and their interaction), type of cerebral palsy (spastic, dyskinetic, ataxic or unclassified), level of dependence (mild, moderate or severe), place of residence (urban versus rural), length of time in the institution (years), and institutionalisation ratio (years divided by age). The model-building process followed a structured approach: these *covariates* were sequentially added to two core models, one including the total St. MQoL-S score and another including all eight domains. Their contribution to model fit was assessed using ANOVA and AIC-guided backward selection [30, 43].

Following model selection, the first core model included the total St. MQoL-S score as the primary explanatory variable, along with age, sex, and cerebral palsy type, selected for their statistical significance and conceptual relevance. For the second core model, five St. MQoL-S domains (self-determination, physical well-being, material well-being, rights and personal development), were retained, along with the same sociodemographic and clinical covariates from the first model [30, 31, 35, 44, 45]. Covariates that did not improve model performance or were not statistically significant were excluded. Although sex was not statistically significant in the stepwise selection process, it was retained as an additional covariate in both core models. This decision was based on its theoretical relevance, its contribution to explaining variation in health utility in previous studies, and its frequent availability in datasets where mapping algorithms are applied [35].

The regression equations of the two core models are represented as follows:

$$\begin{array} {c}{\boldsymbol{E}}{\boldsymbol{Q}}-5{\boldsymbol{D}}\boldsymbol{ }\;{\boldsymbol{i}}{\boldsymbol{n}}{\boldsymbol{d}}{\boldsymbol{e}}{\boldsymbol{x}}=\alpha +{\beta}_{1}*St.MQoL{S}_{Total \;Score} \\ + {\beta }_{2 }*Age+{\beta }_{3 }*Sex+{\beta }_{4 }*CPT \left(Model \;1\right)\\ {\boldsymbol{E}}{\boldsymbol{Q}}-5{\boldsymbol{D}}\boldsymbol{ }\;{\boldsymbol{i}}{\boldsymbol{n}}{\boldsymbol{d}}{\boldsymbol{e}}{\boldsymbol{x}}= \alpha +\sum_{j=1}^{5}{\beta }_{j}*{St.MQoLS}_{{domain}_{j}} \\ + {\beta }_{6 }*Age+{\beta }_{7 }*Sex+{\beta }_{8 }*CPT \left(Model \;2\right)\end{array}$$

where the EQ-5D index is the EQ-5D-5L utility scores and ${St.MQoLS}_{{domain}_{j}}$ represents the St. MQoL-S domains selected according to statistical significance (*p* < 0.05) using stepwise regression.

#### Model types

Three different regression methods were considered for each candidate direct mapping model. First, we employed ordinary least squares *(OLS)* to fit a linear model on the response variable, a method widely used in previous studies with acceptable performance [30, 43, 44]. Despite limitations such as the assumption of normality and the risk of generating predictions outside the valid utility range, OLS remains the most widely used technique for mapping due to its simplicity, transparency, interpretability, parsimony, and ease of replication [46, 47].

Due to the non-normal distribution of the EQ-5D-5L utility scores, and the fact that this variable is constrained to values at or below one, a generalized linear model *(GLM)* with a Gaussian family and a logit link function was chosen as a second candidate. GLMs allow greater flexibility in modelling skewed and bounded outcome variables, which makes them attractive for mapping exercises [30, 34]. Importantly, GLM models have also been applied in previous mapping studies involving populations with CP [31], supporting their relevance for this context. However, GLMs with a logit link function may fail to predict values at the extremes of the utility scale, especially negative utilities, which are relevant in severely disabled populations [48].

The third model considered was a Tobit regression, selected to address the bounded nature of EQ-5D-5L data and the concentration of observations at the upper boundary (utility = 1). Although originally developed for censored outcomes, the Tobit model is widely applied to bounded dependent variables in which values accumulate at the limits [47, 49]. In this setting, it constrains predictions to the theoretical range and can partially mitigate the ceiling effects observed in health-utility data [42]. However, Tobit models do not fully capture other salient features of EQ-5D distributions, such as multimodality, the gap between full health and the next feasible health state, or pronounced skewness. More flexible alternatives, such as beta-based mixture models or the recently proposed adjusted limited dependent variable mixture model (ALDVMM) [47, 50] can be also considered to address some key limitations of the previously described models. However, the small sample size of our study limits the feasibility of applying this modeling approach.

The selection of model types was informed by a comprehensive review of key publications [23, 31, 42, 45, 46] and methodological guidance on utility mapping, including the ISPOR Task Force recommendations [35], the NICE Decision Support Unit Technical Support Documents (TSD 22) [50], and the MAPS reporting standards [34]. Additionally, we drew on the framework developed by Hernández Alava et al. in their comparative analysis of mapping methods for clinical outcomes to preference-based measures [35, 47, 51, 52], which was particularly helpful in assessing the suitability of different models for our sample characteristics. The inclusion of OLS, GLM, and Tobit models reflects both the practical constraints of our data and the need to address the statistical properties of EQ-5D-5L utilities, such as skewness, bounded distribution, and ceiling effects. Comparing these approaches allowed us to evaluate their relative predictive performance and robustness in a real-world sample of adults with CP.

#### Performance of mapping algorithms

Following the guidelines in the literature, we considered several model fit measures to compare the results across different approaches [30, 34]: mean absolute error (MAE), root mean square error (RMSE), and a visual representation of the model fit. Scatter plots were created to illustrate the relationship between the observed and predicted EQ-5D-5L utility scores under the different considered models (see Figs. S2 and S3 in the Supplementary Material). Additionally, empirical cumulative distribution functions (ECDFs) comparing the observed and predicted EQ-5D-5L utility scores are graphically represented in Fig. 5. These functions are useful for detecting potential biases in the predictions of quality of life measures, such as the EQ-5D-5L, as well as identifying outliers that may affect the fit of the mapping model. This is particularly relevant in cost-utility studies, as extreme values can skew the results of the analyses, influencing the conclusions about the cost-utility relationship [23, 47].

### Validation process

Since our objective is to develop a mapping algorithm that can be generalized to other individuals and populations, we also conduct a *cross-validation approach to validate* the predictive performance of the models [53]. Given the absence of a dataset for external validation, internal validation was performed by considering a fivefold cross-validation scheme. We first divide the sample into five random groups. Four groups (80% of the sample) were assigned as the"estimation sample", used for estimating the models regression coefficients, while the remaining group (20% of the sample) form the"validation sample", which was used to evaluate the predicted EQ-5D-5L scores for the various model candidates. This procedure is repeated so that each of the five random groups served as a validation sample. To obtain more reliable and robust estimates of prediction errors, assessed by MAE and RMSE values, a repeated fivefold cross-validation method was implemented.

Data management and all analyses were performed using Microsoft Excel. Statistical analyses were conducted using R software version 4.3.2 [54], and a *mapping calculator was developed* in Excel 2019.

## Results

**Fig. 2:**
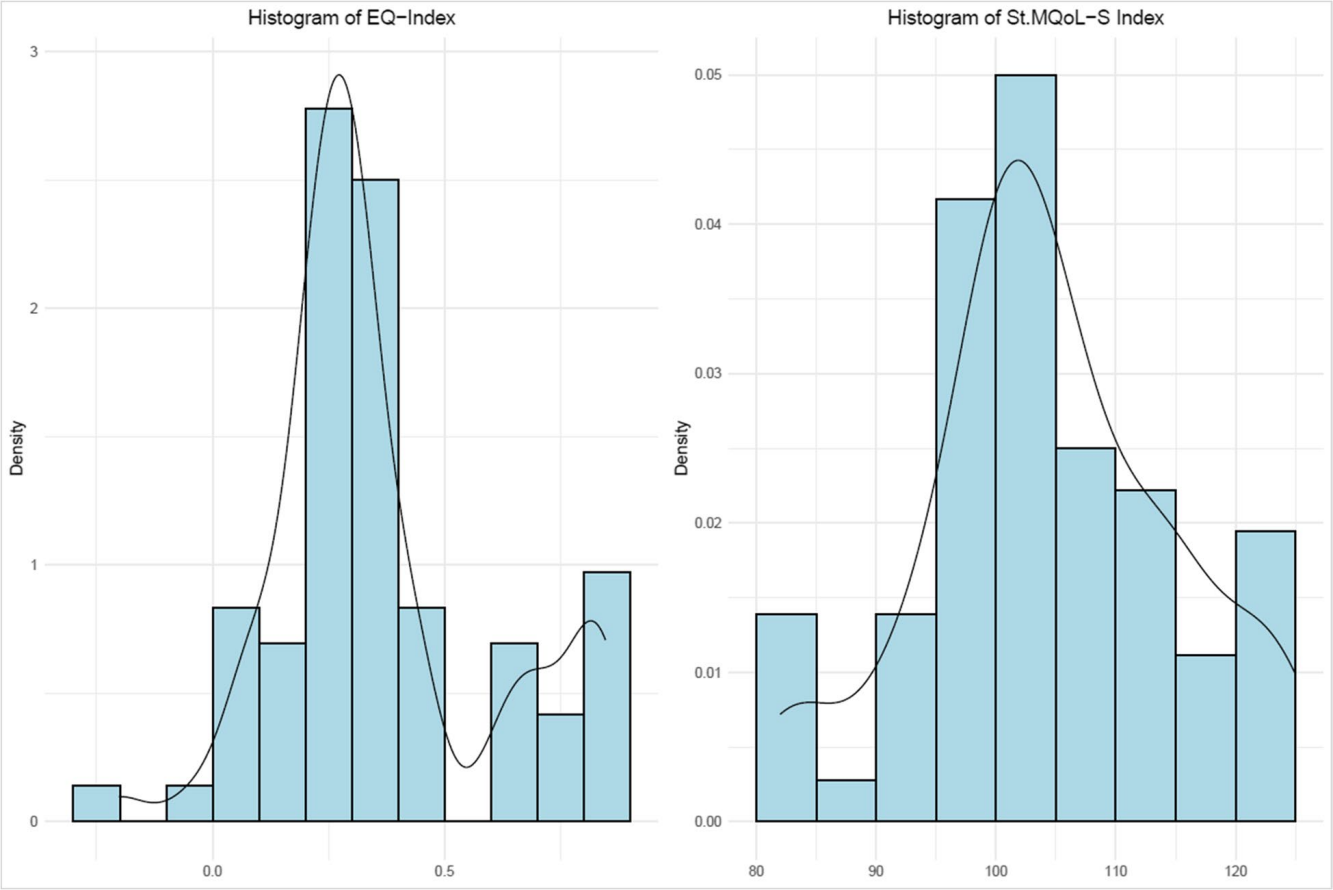
Distribution of EQ-5D-5L utility scores and San Martin Quality of life scale (St. MQoL-S) total scores

Data were collected from 72 participants with CP, who completed both scales at the same time (EQ-5D-5L and the St. MQoL-S). The mean (SD) age was 39.25 (15.77) years. The EQ-5D-5L utility score was not normally distributed (*p* < 0.05). The normality test for the St. MQoL-S total score was not rejected; the distribution was slightly skewed to the left (Fig. 2). The mean (SD) utility of the EQ-5D-5L was 0.36 (0.23), and the mean total score of the St. MQoL-S was 104.40 (10.40). Approximately more than half (61.11%) of the adults were male. Participant characteristics for the entire sample are presented in Table 1.

**Table 1:** Characteristics of the sample (*n* = 72)

**Quantitative variable**	**Mean (SD)**
St. MQoL-S total score	104.40 (10.42)
St. MQoL-S Self-determination domain	12.82 (2.30)
St. MQoL-S Emotional Well-being domain	11.07 (2.17)
St. MQoL-S Physical Well-being domain	10.85 (2.09)
St. MQoL-S Material Well-being domain	10.65 (2.14)
St. MQoL-S Rights domain	11.49 (2.08)
St. MQoL-S Personal Development domain	10.53 (2.70)
St. MQoL-S Interpersonal relationships domain	9.44 (2.48)
St. MQoL-S Social Inclusion domain	9.26 (2.84)
EQ-5D index, Mean (SD)-Spanish tariff	0.36 (0.23)
Age in years, Mean (SD)	39.25 (15.77)
**Qualitative variables (Sociodemographic and Clinical)**	***N* (%)**
Gender	
Female	28 (38.89)
Male	44 (61.11)
Patients living in the main urban area	36 (50.00)
Patients living in the rural area	36 (50.00)
Cerebral palsy type	
Spastic CP	50 (69.44)
Dyskinetic CP	4 (5.56)
Ataxic CP	3 (4.17)
Unclassified CP	15 (20.83)
Functional classification of the recognized level of dependency	
Mild	10 (13.89)
Moderate	16 (22.22)
Severe	46 (63.89)

*CP* Cerebral Palsy, *EQ-5D* index (derived from EQ-5D-5L), *N* Sample size, *SD* standard deviation, *St. MQoL-S* St Martin's Quality of Life Scale

### Assessing the correlation between HRQoL measures

**Fig. 3:**
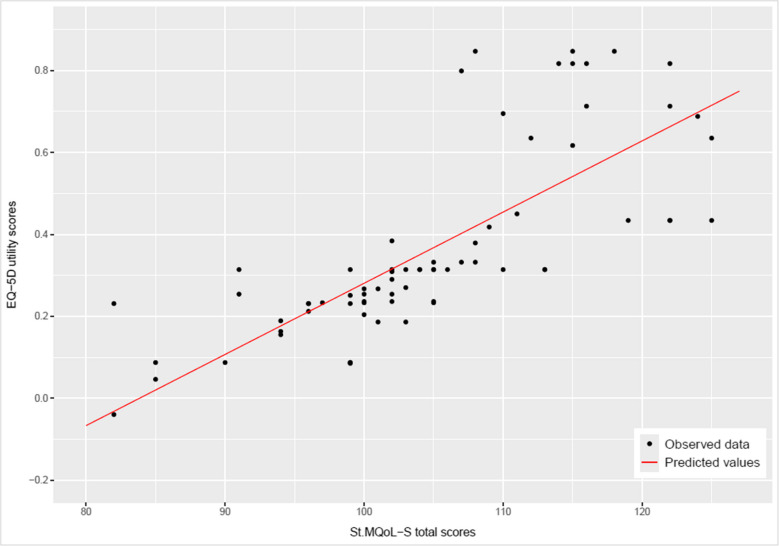
Scatter plot between the EQ-5D utility scores and St. MQoL-S total scores

A strong positive linear correlation was found between the EQ-5D-5L utility scores and the St. MQoL-S total scores (r = 0.77, *p* < 0.05), indicating a significant relationship between the two scales (Fig. 3). At the domain level, high correlations were observed between the utility scores of the EQ-5D-5L and the domains/dimensions of the St. MQoL-S (*p* < 0.05), the Spearman rank correlation ranged from 0.59 to 0.69, suggesting that better predictions would be obtained by considering more dimensions (Table S2 –Fig. S1, supplementary material).

### Mapping model performance

Table 2 presents the goodness-of-fit results of the different models, employing the entirety of the data to estimate the regression coefficients of the explanatory variables. The predicted average utility scores of the EQ-5D-5L are consistently close across the model candidates. Almost all models overestimated the lower bound of the EQ-5D-5L utility scores, while simultaneously underestimating its upper limit.

**Table 2:** Goodness-of-fit results of model candidates

Model specification (*n* = 72)	Differences between predicted and observed mean	Mean predicted	Minimum predicted	Maximum predicted	MAE	RMSE
EQ-5D-5L observed utility	NA	0,3571	−0,202	0,8470	NA	NA
Method 1: Ordinary Least Squares Estimator						
Model 1	0,0000	0,3571	−0,0569	0,7258	0,1000	0,1367
Model 2	0,0000	0,3571	−0,1649	0,7221	0,0990	0,1326
Method 2: Generalized Linear Model: Gaussian family logit link						
Model 1	0,0043	0,3614	0,0650	0,7501	0,0978	0,1319
Model 2	0,0019	0,3590	0,0359	0,7446	**0,0971**	**0,1281**
Method 3: Tobit Estimator						
Model 1	0,0000	0,3571	−0,0569	0,7258	0,1000	0,1367
Model 2	0,0000	0,3571	−0,1649	0,7221	0,0990	0,1326

*EQ-5D-5L* five-level EuroQol five-dimensional questionnaire, *MAE* mean absolute error, *RMSE* Root mean squared error, *NA* not applicable

In terms of goodness-of-fit, GLM model 2 showed the best predictive ability, with the lowest MAE (0.0971) and RMSE (0.1281), followed by GLM model 1 (MAE = 0.0978, RMSE: 0.1319). Additionally, the GLM estimator of model 1 closely predicted the maximum EQ-5D-5L score, while the OLS estimator of model 2, as well as the Tobit estimator of model 2, closely predicted the minimum value.

Regression coefficient estimates for the different model candidates are presented in Table S3 (supplementary material). In model 1, the St. MQoL-S total score was a significant predictor variable (*p* < 0.05) in all three regression methods. In model 2, the self-determination domain on the St. MQoL-S was consistently significant in all three regression methods. The physical well-being domain and the rights domain were significant in the OLS and Tobit estimator, while material well-being was significant in the GLM and Tobit estimator. The personal development domain was significant only in the Tobit estimator. Age was significant in the GLM and Tobit estimator (*p* < 0.1) and type of CP was also a significant predictor (*p* < 0.05).

It should be recalled that all the variables mentioned in Table S3* were significant in the ANOVA F-test when selecting the explanatory variables for predicting the EQ-5D-5L utility score.* This was reflected in the variables that were retained in the two core models described above in the statistical analysis, in order to obtain the best possible mapping algorithm. Although the sex variable was not statistically significant, we decided to include it due to its potential to enhance the mapping algorithm and its recognized importance as a covariate in other populations.

### Validation of mapping algorithms

Although GLM model 2 was initially identified as the best model to predict EQ-5D-5L utilities from the St. MQoL-S scores and the explanatory variables within our observed data, a cross-validation approach has been conducted to ensure generalization of this finding for out-of-sample individuals. Table 3 displays the result obtained under a repeated 5-fold cross-validation scheme.

**Table 3:** Goodness of fit results using a repeated 5-fold cross-validation scheme

Model specification (repeated 5-fold CV)	Differences between predicted and observed mean	Mean predicted	Minimum predicted	Maximum predicted	MAE	RMSE
EQ-5D-5L observed utility	NA	0,3571	−0,202	0,8470	NA	NA
Method 1: Ordinary Least Squares Estimator						
Model 1	0,0019	0,3590	−0,0986	0,8703	**0,1155**	**0,1567**
Model 2	0,0016	0,3587	−0,1918	0,8773	0,1273	0,1683
Method 2: Generalized Linear Model: Gaussian family logit link						
Model 1	0,0030	0,3601	0,0541	0,8140	0,1215	0,1672
Model 2	0,0060	0,3631	0,0310	0,8776	0,1258	0,1663
Method 3: Tobit Estimator						
Model 1	0,0002	0,3573	−0,0901	0,8866	0,1140	0,1562
Model 2	0,0004	0,3575	−0,1904	0,9148	0,1230	0,1651

*EQ-5D-5L* five-level EuroQol five-dimensional questionnaire, *MAE* mean absolute error, *RMSE* Root mean squared error, *NA* not applicable

**Fig. 4:**
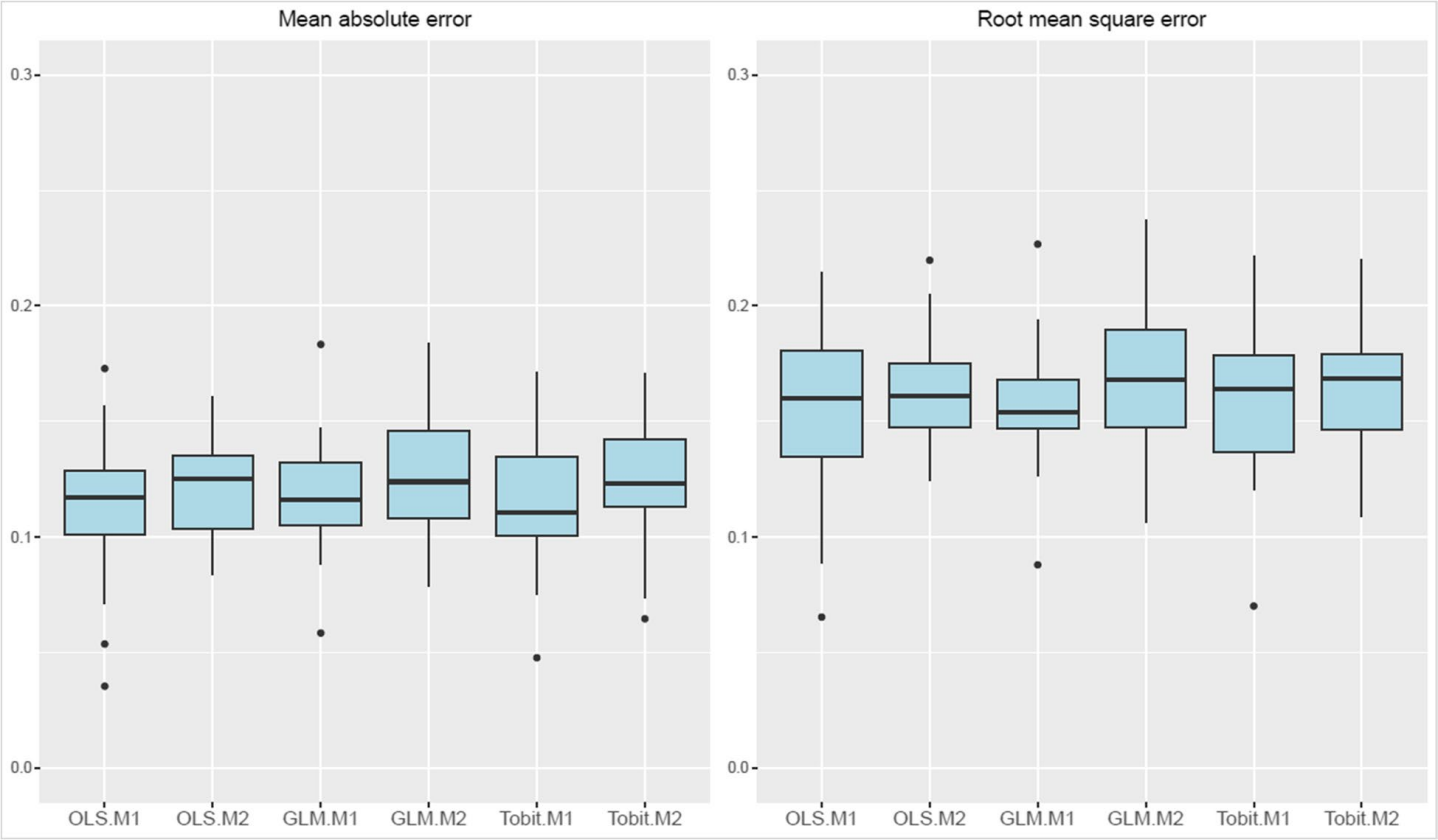
Box plots of estimated prediction error measures among different cross-validation samples

**Fig. 5:**
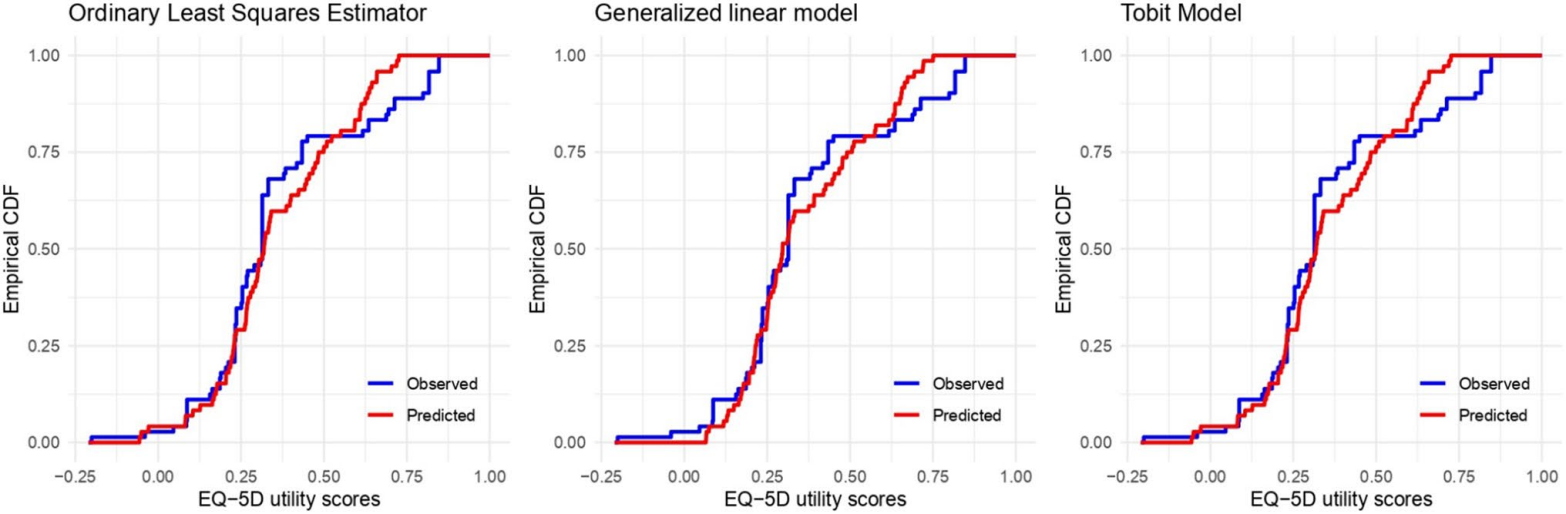
Empirical cumulative distribution functions from the real data. OLS regression model, GLM regression model and Tobit regression model

According to this analysis, the OLS model 1 exhibits the best predictive performance with lowest MAE (0.1131) and RMSE (0.1545) values. This suggests its preference as the mapping algorithm of choice for applications in other populations that have not collected utility data. However, overall differences between OLS, GLM, and Tobit models were small (Fig. 4). The scatter plots for the observed and predicted EQ-5D utility scores of all mapping models are shown in Figs. S2 and S3 (supplementary material). Furthermore, Fig. 5 demonstrates that the Tobit and OLS models show slightly less bias compared to the GLM model, providing a closer fit to the data, particularly at both low and high values of the EQ-5D index.

Despite the slightly better in-sample fit observed for GLM model 2, OLS model 1 was ultimately selected as the preferred algorithm. This decision was based on its superior predictive performance under our cross-validation study, which better reflects out-of-sample generalizability. In addition, OLS is widely used in utility mapping studies due to its simplicity, transparency, and ease of implementation. Its coefficients are easily interpretable, and the method is readily reproducible across datasets without requiring specialised software, making it especially suitable for use by researchers, clinicians, and health technology assessment agencies. These practical advantages, combined with its robust out-of-sample accuracy, support the selection of OLS model 1 for predicting EQ-5D-5L utilities in adults with CP.

The *preferred mapping algorithm*, represented through the *OLS 1 model*, can be presented as follows:

$$\begin{aligned} & {\boldsymbol{E}}{\boldsymbol{Q}}-5{\boldsymbol{D}}\boldsymbol{ }\;{\boldsymbol{i}}{\boldsymbol{n}}{\boldsymbol{d}}{\boldsymbol{e}}{\boldsymbol{x}}=-1.3800+0.0157 \\& *St.MQoL{S}_{Total \;Score}+ 0.0018*Age \\& +0.0016*I \left(Sex=Male\right)+0.0775*I \\& \left(CPT=Dyskinetic\right)+0.1658*I(CPT=Ataxic) \\& +0.0842*I(CPT=Unclassified) \end{aligned}$$

where the symbol I (·) denotes an indicator function.

After obtaining the mapping algorithm that best fits the data, the QALY per year for an adult person with CP can be obtained. For example, a person who has a St. MQoL-S total score of 106, is 30 years old, is female and has a CP of the spastic type, her score would be:

$${{\boldsymbol{Q}}{\boldsymbol{A}}{\boldsymbol{L}}{\boldsymbol{Y}}}_{{\boldsymbol{P}}{\boldsymbol{e}}{\boldsymbol{r}}\boldsymbol{ }{\boldsymbol{y}}{\boldsymbol{e}}{\boldsymbol{a}}{\boldsymbol{r}}}=-1.3800+0.0157*(106)+ 0.0018*30 = 0.3382$$

### Uncertainty of individual-level predictions

Table S4 (supplementary material), includes the observed and predicted utility scores based on the best-performing OLS model 1, along with their 95% confidence intervals at the individual level. Including confidence intervals for individual-level predictions is crucial because they provide a measure of the uncertainty associated with each estimate. These intervals help to quantify the potential variability in predicted outcomes, allowing clinicians and researchers to assess the reliability of the predictions. This observed variability emphasizes the necessity of considering these ranges when interpreting the results, as they can significantly influence clinical decisions and economic evaluations. This will allow users of this mapping algorithm to estimate standard errors around mean utility predictions and assess variability at the individual level.

The covariance matrix for the model was used to allow for variability and correlations between variables. The covariance matrices obtained for the OLS, GLM and Tobit model 1 exhibit low multicollinearity among the explanatory variables, suggesting that they contribute independently to the mapping model. Furthermore, significant relationships are observed in the GLM model, particularly between the St. MQoL Index and variables such as gender and types of CP, supporting the ability of these variables to capture significant differences in the prediction of the EQ-5D index. These observations reinforce the statistical validity of the mapping between the St. MQoL-S and the EQ-5D-5L in this population of adult patients with CP. The full covariance matrices for each model are included in the supplementary material (Table S5).

### Methodological reproducibility and replicability

To facilitate the replicability and use of the proposed mapping algorithm, the original data and R code used in this analysis are available as supplementary material (see Data_and_Rcode.zip). Researchers can refer to the attached README file, which provides a comprehensive description of the data and detailed R scripts to obtain the best mapping algorithm between St. MQoL-S and EQ-5D-5L. Specifically, the code is organized into four scripts that reproduce the main steps of our analysis: descriptive analysis, variable selection, mapping model performance, and the cross-validation study. This not only allows them to run the code to calculate EQ-5D-5L utility values from St. MQoL-S scores, but also to gain a deeper understanding of the methodology employed. Consulting these additional resources enhances the critical appraisal of the results, ensures greater transparency in the methodological process, and provides examples illustrating the use of the mapping algorithm in the corresponding section of the README file.

## Discussion

Our study provides evidence that the St. MQoL-S could conceptually serve as a promising candidate for mapping to the EQ-5D-5L in adult patients with CP. This could be partly explained by the fact that our mapping results fall within the MAE and RMSE ranges of other mapping studies [23, 44, 46] and allowed us to affirm the viability of mapping from the St. MQoL-S to the EQ-5D-5L. Our mapping models showed significantly better predictive outcomes when utilizing the St. MQoL-S total score/index as an explanatory variable instead of scores from the most significant dimensions (Self-determination, Physical wellbeing, Material well-being, Rights and Personal development) when estimating EQ-5D-5L utilities. This enhancement can be attributed to the robust correlation observed between the total scores of the two HRQoL scales and the specific characteristics of the study population [31, 42].

In line with prior mapping studies, our cross-validation results confirmed that OLS model 1 achieved the highest predictive accuracy The Tobit model also showed competitive performance and may be particularly well-suited for mapping bounded outcome variables [42, 47]. Although GLM model 2 exhibited marginally better in-sample fit, it was consistently outperformed by OLS model 1 in predictive performance. Additionally, the GLM models with logit link functions showed slightly poorer overall accuracy, as they are unable to predict negative EQ-5D-5L utility values, resulting in higher error rates for observations with low utility scores. The preference for OLS is grounded in its simplicity, transparency, and ease of implementation. These advantages are especially valuable in small samples, where more complex models may risk overfitting and may be harder to replicate by external users. Additionally, OLS is commonly used in mapping studies, and its adoption facilitates comparability across evaluations [34, 50].

Our preferred OLS model 1, which included the total score of the St. MQoL-S along with age, sex, and CP type, yielded MAE and RMSE values consistent with those found in the neurological and general mapping literature [31, 42, 55]. The contrast between in-sample fit and cross-validated performance underlines the importance of validation techniques for selecting robust mapping algorithms [30, 35]. The mapping methodology offers a valuable tool for integrating, comparing and synthesizing data from different sources and health outcome measures to obtain QALYs in cost effectiveness analyses [56]. This technique facilitates the extrapolation of data when health utility data are not collected directly, thus allowing a more complete estimation of the impact of health interventions related to the HRQoL [34, 43, 45] of patients with pathologies as specific as cerebral palsy [31]. In addition, mapping enables direct comparison of the relative effectiveness and efficiency of different intervention strategies, which is essential for informed decision making in health policy and resource allocation in public health [30, 35].

This study represents a meaningful contribution to the sparse literature on economic evaluation in adult CP populations. Previous mapping studies in CP populations have been limited in number [31, 43]. To our knowledge, it is the first study to propose a mapping algorithm between the St. MQoL-S and EQ-5D-5L specifically in adults with CP. An important strength of the analysis lies in the integrity of the returned questionnaires, with no evidence of missing data, as patients were always accompanied when responding to questionnaires to clarify doubts, enable simultaneous completion of both questionnaires, and avoid unanswered questions. The proposed algorithm has practical implications for clinicians, researchers, and policymakers. It enables reliable estimation of EQ-5D-5L utility values from St. MQoL-S data, supporting cost-utility analyses even when preference-based utility measures are not available. This is particularly relevant for health technology assessments in Spain, where the EQ-5D is the recommended instrument for measuring utilities.

## Limitations

This study has some limitations that should be acknowledged. First, the relatively small sample size (*n* = 72) constitutes a major limitation that introduces notable uncertainty in the estimated EQ-5D-5L utility values. Small samples increase the risk of overfitting, reduce the stability of parameter estimates, and limit the generalizability of results to broader cerebral palsy populations [50]. Therefore, while the present mapping function may serve as a useful preliminary tool, its outputs should be interpreted with caution in applied settings. Given the limited availability of data sources for adults with cerebral palsy, particularly in Spain [39], this study offers a necessary first step. Future research should focus on validating these findings in larger and more diverse samples to enhance external validity and reduce uncertainty in health economic applications.

Second, generalisability to other populations warrants careful consideration. Although our sample is collected in Spain, the St. MQoL-S is an internationally validated instrument, culturally adapted into several languages (e.g., English, French, Italian, Portuguese, Hungarian), which enhances its potential applicability in other countries [22, 57]. Moreover, it has been validated in populations with conditions frequently comorbid with CP, including epilepsy, autism spectrum disorder, sensory impairments, and behavioural and emotional disorders, supporting the plausibility of the mapping algorithm in related subgroups [18, 21]. However, the instrument has also been validated in people with Down syndrome, whose distinct etiology and clinical progression suggest caution when extending our model to this population. Future work should assess external validity in international CP cohorts and, where justified, in broader disability groups sharing overlapping features.

Third, and consistent with findings in other mapping studies, our models exhibited systematic bias at the boundaries of the EQ-5D-5L utility scale. Specifically, there is a tendency to overestimate low utility values and underestimate high ones [48, 51]. This issue is especially important in the context of CP, a lifelong disabling condition often associated with low utility scores. Overestimating these values could lead to an underestimation of the true benefit of interventions targeting individuals in poor health states, which may bias cost-effectiveness results and subsequent funding decisions. While flexible methods such as *splines* or adjusted limited dependent variable mixture models (ALDVMM) may reduce this bias by better capturing the multimodal and bounded distribution of EQ-5D-5L utilities, their use generally requires larger datasets and more complex modelling strategies than were feasible in the present study [47, 58]. In our comparison (results not shown here), the ALDVMM demonstrated inferior model fit compared to the other mapping algorithms presented in this paper. Moreover, due to the limited sample size of our data, incorporating the ALDVMM into our validation study was not feasible.

Despite its limitations, this study provides a meaningful contribution to health economic evaluation in adults with cerebral palsy, a population often underrepresented in the health technology assessment literature. The proposed mapping algorithm offers a pragmatic and replicable solution for estimating utility values in the absence of preference-based measures, laying the foundation for further research and policy use.

## Conclusions

Several studies focusing on quality of life in populations affected by CP have dispensed with the use of preference-based utility measures, despite their increasing need to inform decisions in economic evaluations needed to assess the quality of treatments in CP. The algorithms presented in this study offer an alternative for estimating the utility of the EQ-5D-5L in scenarios where utility information has not been directly collected from CP patients. Our research has demonstrated the feasibility of predicting, with reasonable accuracy (based on MAE and RMSE results reported in other mapping studies), the utility values of the EQ-5D-5L derived from the St. MQoL-S. These findings should help the economic evaluations of interventions for adults with CP by providing evidence linking the St. MQoL-S, an outcome measure of interventions aimed at the specialized care of adults with CP used in different countries, to a generic preference-based tool that is widely used around the world, the EQ-5D-5L.

To our knowledge, this is the first study to map from St. MQoL-S to the preference-based measure EQ-5D-5L, the preferred measure for economic evaluation. The paper presents mapping functions to generate utility values from St. MQoL-S to EQ-5D-5L. When only point estimates are considered, there is little difference between the different mapping methods. However, our cross-validation study confirms that the mapping represented by the OLS model 1 outperforms both the Tobit and GLM mapping methods. Therefore, we recommend using the OLS model 1 mapping technique to generate utilities for cost-utility analysis. *We have created a tool in the form of a calculator to assist researchers* in easily calculating predicted utilities from St. MQoL-S. In the future, it will be necessary to compare the values generated from larger samples for indirect mapping and allocation algorithms with those derived directly from the new set of preference weights obtained using the St. MQoL-S domain items.

## Electronic supplementary material

Below is the link to the electronic supplementary material.

Supplementary file1 (DOCX 298 KB)

Supplementary file2 (XLSM 1249 KB)

Supplementary file3 (XLSX 16 KB)

Supplementary file4 (R 6 KB)

Supplementary file5 (R 3 KB)

Supplementary file6 (R 3 KB)

Supplementary file7 (R 3 KB)

Supplementary file8 (R 11 KB)

Supplementary file9 (R 8 KB)

Supplementary file10 (HTML 27 KB)

Supplementary file11 (R 15 KB)

## Data Availability

The data and codes used to obtain the results of this manuscript are available in the supplementary material attached to this manuscript, within a compressed zip file (Data_and_Rcode.zip). This file includes a README link that provides access to the data and instructions for replicating the code.
