# Supplementary material for: Estimating QALYs in adults with cerebral palsy: mapping the San Martin scale to the EQ-5D-5L for economic evaluation
Source: Eur J Health Econ. 2025 Sep 24;27(3):527–40. doi: 10.1007/s10198-025-01831-1 (PMC13190523; doi:10.1007/s10198-025-01831-1)

**Supplementary Material**

**Table S1: Comparison between the domains of the scales: St. MQoL-S and EQ-5D-5L**

| **EQ-5D-5L domains** | **San Martin Quality of Life Scale (St. MQoL-S)** | |
| --- | --- | --- |
|  | **Items** | **Domains** |
| **Mobility** | Specific measures are taken in relation to their mobility in order to encourage their independence | Physical well-being |
|  |  |  |
|  | Moves through different spaces (i.e., indoors and outdoors) |  |
|  | Specific measures are taken to prevent or treat problems arising from physical disabilities (e.g., spasticity, stiffness, deformity, etc.) |  |
| **Selfcare** | Specific measures are taken by the institution to avoid risks such as blows, falls and escapes. | Material well-being |
|  | Specific measures are taken to adapt the living environment to the person's abilities and limitations (i.e. sensory, cognitive, behavioural, physical). |  |
|  | Chooses the meal or part of the meal when there is variety in 1st, 2nd and dessert. |  |
|  | Has a daily schedule of activities appropriate to his/her preferences |  |
|  | They have a personal record of what they like, what calms them, what they cannot tolerate and how they may react, which all staff are aware of and must comply with. | Emotional well-being |
|  | Appropriate love, affection and physical contact are provided when needed. |  |
|  | Participates in inclusive activities appropriate to their physical and mental condition | Social Inclusion |
|  | Has adequate hygiene (e.g., teeth, hair, nails, body) and personal image (e.g., age-appropriate clothing, occasion-appropriate clothing, etc.). (e.g., clothing appropriate to age, occasion, etc. | Physical well-being |
| **Usual activities** | You choose how you spend your free/leisure time | Self-determination |
|  | Has opportunities to refuse to do activities that are irrelevant to his/her health |  |
|  | You choose the meal and when want to eat |  |
|  | Has a daily schedule of activities appropriate to his/her preferences |  |
|  | Performs physical activities and exercises appropriate to his or her characteristics and needs. | Physical well-being |
|  | You have a programme of activities with things that you enjoy and contribute to your personal enrichment. | Personal Development/Rights |
|  | The activities you do allow you to learn new skills. |  |
|  | You learn things that make you more independent |  |
|  | At the centre you have opportunities to show your skills |  |
|  | You gain new skills or experiences by participating in activities. |  |
|  | Participates in activities outside the centre with people outside his/her support context. | Social Inclusion |
|  | The activities in which he/she participates consider the leisure and cultural facilities in the area. |  |
|  | Specific measures are taken to offer as wide a variety of activities as possible (e.g., new activities based on people's preferences). |  |
|  | Participates in inclusive activities that interest him/her |  |
|  | Participates in social activities outside the place where he/she receives services or supports |  |
| **Pain/Discomfort** | Specific measures are taken to prevent or treat pain | Physical well-being |
|  | Special attention is given to the diagnosis and treatment of any sensory disabilities you may have. |  |
|  |  |  |
| **Anxiety/Depression** | Your individual expressions of distress are known to the people who support you. | Emotional well-being |
|  | Supporters pay attention to your facial expressions, glances and eye direction, eyesight facial expressions, gaze and eye direction, voice, muscle tension, posture, movement, and physiological reactions. |  |
|  | Supporters know how you express your wishes. |  |
| For more detailed information on the San Martín Quality of Life Scale and EQ-5D-5L instrument please consult the following link:  <https://sid-inico.usal.es/idocs/F8/FDO26729/San_Martin_Scale_English_(Verdugo_Gomez_et_al_2014).pdf>  <https://euroqol.org/information-and-support/euroqol-instruments/eq-5d-5l/> | | |

|  | EQ-5D utility scores | St. MQoL-S Self- determination domain | St. MQoL-S Emotional Well-being domain | St. MQoL-S Physical Well-being domain | St. MQoL-S Material Well-being domain | St. MQoL-S Rights domain | St. MQoL-S  Personal Developmental domain | St. MQoL-S  Interpersonal Relationships domain | St. MQoL-S  Social Inclusion  domain |
| --- | --- | --- | --- | --- | --- | --- | --- | --- | --- |
| St. MQoL-S Self-determination domain | 0.68* |  |  |  |  |  |  |  |  |
| St. MQoL-S Emotional Well-being domain | 0.55* | 0.47* |  |  |  |  |  |  |  |
| St. MQoL-S Physical Well-being domain | 0.65* | 0.35* | 0.43* |  |  |  |  |  |  |
| St. MQoL-S Material Well-being domain | 0.60* | 0.45* | 0.48* | 0.56* |  |  |  |  |  |
| St. MQoL-S Rights domain | 0.59* | 0.26* | 0.53* | 0.61* | 0.55* |  |  |  |  |
| St. MQoL-S Personal Developmental domain | 0.66* | 0.50* | 0.61* | 0.47* | 0.49* | 0.56* |  |  |  |
| St. MQoL-S Interpersonal Relationships  domain | 0.67* | 0.43* | 0.58* | 0.65* | 0.55* | 0.59* | 0.70* |  |  |
| St. MQoL-S Social  Inclusion  domain | 0.67* | 0.49* | 0.15 | 0.43* | 0.24* | 0.18 | 0.37* | 0.35* |  |

**Table S2: Spearman correlation coefficients between EQ-5D utility score and domains of St. MQoL-S total scores**

*Note * p-value <0.05*

**Table S3: Estimated regression coefficients of model candidates**

| **Independent variables** | **Ordinary Least Squares estimator** | **Generalized Linear Model** | **Tobit Model** |
| --- | --- | --- | --- |
| *Panel A - Model 1* |  |  |  |
| St. MQoL-S total scores | 0.016 (0.002) **** | 0.075 (0.010) **** | 0.016 (0.002) **** |
| Age (year) | 0.002 (0.001) | 0.010 (0.006) * | 0.002 (0.001) * |
| Sex | 0.002 (0.035) | 0.007 (0.174) | 0.002 (0.034) |
| CP Dyskinetic | 0.078 (0.078) | 0.293 (0.328) | 0.078 (0.074) |
| CP Ataxic | 0.166 (0.088) * | 0.604 (0.378) | 0.016 (0.084) ** |
| CP Unclassified | 0.084 (0.043) * | 0.390 (0.200) * | 0.084 (0.041) ** |
| Constant | -1.380 (0.178) **** | -9.041 (1.031) **** | -1.380 (0.169) **** |
| *Panel B - Model 2* |  |  |  |
| St. MQoL-S  Self-determination domain | 0.025 (0.010) ** | 0.131 (0.048) *** | 0.025 (0.009) *** |
| St. MQoL-S  Physical Well-Being domain | 0.020 (0.011) * | 0.091 (0.055) | 0.020 (0.010) * |
| St. MQoL-S  Material Well-Being domain | 0.018 (0.012) | 0.104 (0.054) * | 0.018 (0.011) * |
| St. MQoL-S  Rights domain | 0.022 (0.013) * | 0.107 (0.070) | 0.022 (0.012) * |
| St. MQoL-S  Personal Development domain | 0.014 (0.009) | 0.058 (0.041) | 0.014 (0.008) * |
| Age (year) | 0.001 (0.001) | 0.006 (0.006) | 0.001 (0.001) |
| Sex | -0.005 (0.037) | -0.040 (0.184) | -0.005 (0.034) |
| CP Dyskinetic | 0.100 (0.081) | 0.422 (0.345) | 0.100 (0.074) |
| CP Ataxic | 0.188 (0.089) ** | 0.712 (0.389) * | 0.188 (0.082) ** |
| CP Unclassified | 0.070 (0.044) | 0.302 (0.206) | 0.070 (0.041) |
| Constant | -0.838 (0.126) **** | -6.651 (0.799) **** | -0.838 (0.116) **** |
| CP Spastic=1, CP Dyskinetic=2, CP Ataxic=3, CP Unclassified=4  Model 1 uses total St. MQoL-S scores to predict EQ5D utility scores;  Model 2 uses St. MQoL-S domain scores to predict EQ5D utility scores  Sex= Female=0, Male=1  **** Significant at p<0.001; ***Significant values at p<0.01; **Significant values at p<0.05; * Significant values at p<0.1  Standard errors in parentheses  Model coefficients were rounded to three digits. | | | |

**Table S4. Predicted EQ-5D utility score and 95% confidence interval for the OLS model 1**

| **St. MQoL-S total scores** | **Age (years)** | **Sex** | **CP Type** | **Observed EQ-5D utility score** | **Predicted EQ-5D utility score** | **95% confidence interval** |
| --- | --- | --- | --- | --- | --- | --- |
| 82 | 20 | Male | Spastic | -0.202 | -0.057 | [-0.151, 0.037] |
| 82 | 35 | Male | Spastic | -0.040 | -0.030 | [-0.121, 0.061] |
| 85 | 72 | Female | Spastic | 0.046 | 0.082 | [-0.038, 0.202] |
| 99 | 18 | Female | Spastic | 0.085 | 0.204 | [0.132, 0.277] |
| 85 | 25 | Female | Unclassified | 0.087 | 0.082 | [-0.031, 0.195] |
| 90 | 52 | Male | Spastic | 0.087 | 0.126 | [0.045, 0.208] |
| 99 | 49 | Male | Spastic | 0.087 | 0.262 | [0.201, 0.323] |
| 99 | 29 | Male | Spastic | 0.087 | 0.226 | [0.17,0 0.282] |
| 94 | 60 | Female | Unclassified | 0.155 | 0.286 | [0.177, 0.395] |
| 94 | 47 | Male | Unclassified | 0.163 | 0.264 | [0.175, 0.354] |
| 101 | 18 | Female | Spastic | 0.186 | 0.236 | [0.163, 0.308] |
| 103 | 18 | Male | Spastic | 0.186 | 0.269 | [0.202, 0.336] |
| 94 | 76 | Female | Spastic | 0.189 | 0.231 | [0.120, 0.341] |
| 100 | 18 | Male | Spastic | 0.204 | 0.222 | [0.155, 0.288] |
| 96 | 24 | Female | Spastic | 0.212 | 0.168 | [0.101, 0.235] |
| 96 | 29 | Female | Spastic | 0.231 | 0.177 | [0.114, 0.240] |
| 99 | 26 | Female | Spastic | 0.231 | 0.219 | [0.156, 0.282] |
| 96 | 49 | Female | Spastic | 0.231 | 0.213 | [0.147, 0.280] |
| 82 | 23 | Male | Spastic | 0.231 | -0.051 | [-0.144, 0.041] |
| 100 | 44 | Female | Spastic | 0.233 | 0.267 | [0.208, 0.326] |
| 97 | 18 | Male | Spastic | 0.233 | 0.175 | [0.106, 0.243] |
| 105 | 18 | Male | Spastic | 0.233 | 0.300 | [0.232, 0.368] |
| 105 | 54 | Male | Dyskinetic | 0.236 | 0.443 | [0.295, 0.590] |
| 102 | 66 | Female | Spastic | 0.236 | 0.338 | [0.253, 0.423] |
| 100 | 21 | Male | Spastic | 0.236 | 0.227 | [0.164, 0.290] |
| 99 | 20 | Male | Unclassified | 0.251 | 0.294 | [0.205, 0.382] |
| 102 | 28 | Male | Spastic | 0.254 | 0.271 | [0.216, 0.326] |
| 91 | 31 | Male | Spastic | 0.254 | 0.104 | [0.037, 0.171] |
| 100 | 23 | Male | Spastic | 0.254 | 0.231 | [0.170, 0.291] |
| 101 | 54 | Male | Spastic | 0.267 | 0.302 | [0.237, 0.368] |
| 100 | 25 | Male | Unclassified | 0.267 | 0.318 | [0.235, 0.402] |
| 103 | 57 | Male | Unclassified | 0.270 | 0.423 | [0.335, 0.511] |
| 102 | 25 | Male | Spastic | 0.290 | 0.266 | [0.208, 0.324] |
| 102 | 56 | Male | Spastic | 0.309 | 0.322 | [0.254, 0.389] |
| 102 | 32 | Male | Spastic | 0.314 | 0.278 | [0.226, 0.331] |
| 110 | 45 | Male | Ataxic | 0.314 | 0.593 | [0.425, 0.761] |
| 104 | 49 | Male | Spastic | 0.314 | 0.340 | [0.283, 0.398] |
| 103 | 38 | Female | Spastic | 0.314 | 0.303 | [0.246, 0.360] |
| 104 | 35 | Female | Spastic | 0.314 | 0.313 | [0.256, 0.371] |
| 105 | 38 | Female | Spastic | 0.314 | 0.334 | [0.277, 0.392] |
| 99 | 18 | Female | Spastic | 0.314 | 0.204 | [0.132, 0.277] |
| 105 | 18 | Female | Spastic | 0.314 | 0.298 | [0.224, 0.373] |
| 106 | 20 | Male | Spastic | 0.314 | 0.319 | [0.254, 0.385] |
| 113 | 18 | Female | Unclassified | 0.314 | 0.508 | [0.397, 0.619] |
| 91 | 18 | Female | Unclassified | 0.314 | 0.163 | [0.054, 0.272] |
| 113 | 49 | Male | Spastic | 0.314 | 0.481 | [0.418, 0.545] |
| 107 | 50 | Male | Unclassified | 0.332 | 0.473 | [0.393, 0.554] |
| 108 | 40 | Male | Spastic | 0.332 | 0.387 | [0.333, 0.440] |
| 105 | 37 | Female | Spastic | 0.332 | 0.333 | [0.275, 0.390] |
| 108 | 47 | Female | Spastic | 0.379 | 0.398 | [0.336, 0.460] |
| 102 | 58 | Female | Spastic | 0.384 | 0.324 | [0.251, 0.396] |
| 109 | 39 | Male | Spastic | 0.418 | 0.401 | [0.346, 0.455] |
| 122 | 41 | Male | Spastic | 0.434 | 0.608 | [0.527, 0.689] |
| 122 | 56 | Male | Unclassified | 0.434 | 0.719 | [0.620, 0.819] |
| 125 | 36 | Female | Spastic | 0.434 | 0.644 | [0.546, 0.743] |
| 119 | 23 | Male | Unclassified | 0.434 | 0.613 | [0.512, 0.713] |
| 111 | 59 | Female | Spastic | 0.450 | 0.466 | [0.390, 0.543] |
| 115 | 72 | Male | Dyskinetic | 0.617 | 0.632 | [0.481, 0.783] |
| 112 | 39 | Male | Spastic | 0.635 | 0.448 | [0.389, 0.506] |
| 125 | 32 | Female | Spastic | 0.635 | 0.637 | [0.536, 0.738] |
| 124 | 52 | Male | Spastic | 0.688 | 0.659 | [0.570, 0.748] |
| 110 | 34 | Female | Dyskinetic | 0.695 | 0.483 | [0.324, 0.642] |
| 122 | 51 | Male | Dyskinetic | 0.713 | 0.704 | [0.556, 0.852] |
| 116 | 62 | Female | Spastic | 0.713 | 0.550 | [0.464, 0.637] |
| 107 | 40 | Male | Unclassified | 0.799 | 0.455 | [0.379, 0.532] |
| 115 | 47 | Male | Unclassified | 0.817 | 0.593 | [0.509, 0.678] |
| 114 | 47 | Male | Ataxic | 0.817 | 0.659 | [0.492, 0.827] |
| 116 | 47 | Male | Unclassified | 0.817 | 0.609 | [0.523, 0.695] |
| 122 | 50 | Male | Spastic | 0.817 | 0.624 | [0.541, 0.707] |
| 115 | 55 | Male | Spastic | 0.847 | 0.524 | [0.451, 0.596] |
| 108 | 56 | Male | Unclassified | 0.847 | 0.500 | [0.414, 0.585] |
| 118 | 50 | Female | Ataxic | 0.847 | 0.726 | [0.553, 0.899] |

**Table S5. Estimates of the covariance matrices of the regression coefficients of the models (without the intercept).**

| **OLS - M1*** | **STMartin.INDEX** | **AGE** | **SEX1** | **CPT2** | **CPT3** | **CPT4** |
| --- | --- | --- | --- | --- | --- | --- |
| **STMartin.INDEX** | 3.0e-06 | 0.0e+00 | -0.000005 | -0.000024 | -0.000030 | -0.000004 |
| **AGE** | 0.0e+00 | 1.0e-06 | 0.000002 | -0.000016 | -0.000090 | -0.000002 |
| **SEX1** | -5.0e-06 | 2.0e-06 | 0.001251 | -0.000217 | -0.000097 | -0.000211 |
| **CPT2** | -2.4e-05 | -1.6e-05 | -0.000217 | 0.006111 | 0.000857 | 0.000522 |
| **CPT3** | -3.0e-05 | -9.0e-06 | -0.000097 | 0.000857 | 0.007729 | 0.000499 |
| **CPT4** | -4.0e-06 | -2.0e-06 | -0.000211 | 0.000522 | 0.000499 | 0.001840 |
| **GLM - M1*** | **STMartin.INDEX** | **AGE** | **SEX1** | **CPT2** | **CPT3** | **CPT4** |
| **STMartin.INDEX** | 0.000095 | -0.000007 | -0.000178 | -0.000405 | -0.000572 | -0.000012 |
| **AGE** | -0.000007 | 0.000032 | 0.000000 | -0.000335 | -0.000165 | -0.000018 |
| **SEX1** | -0.000178 | 0.000000 | 0.003028 | -0.003962 | -0.004414 | -0.007850 |
| **CPT2** | -0.000405 | -0.000335 | -0.003962 | 0.107410 | 0.016102 | 0.011873 |
| **CPT3** | -0.000572 | -0.000165 | -0.004414 | 0.016102 | 0.142829 | 0.011985 |
| **CPT4** | -0.000012 | -0.000018 | -0.007850 | 0.011873 | 0.011985 | 0.040199 |
| **Tobit - M1*** | **STMartin.INDEX** | **AGE** | **SEX1** | **CPT2** | **CPT3** | **CPT4** |
| **STMartin.INDEX** | 3.0e-06 | 0.0e+00 | -0.000005 | -0.000022 | -0.000027 | -0.000004 |
| **AGE** | 0.0e+00 | 1.0e-06 | 0.000002 | -0.000015 | -0.000088 | -0.000006 |
| **SEX1** | -5.0e-06 | 2.0e-06 | 0.001960 | -0.000196 | -0.000088 | -0.000190 |
| **CPT2** | -2.2e-05 | -1.5e-05 | -0.000196 | 0.005519 | 0.000774 | 0.000471 |
| **CPT3** | -2.7e-05 | -8.0e-06 | -0.000088 | 0.000774 | 0.006984 | 0.000451 |
| **CPT4** | -4.0e-06 | -2.0e-06 | -0.000190 | 0.000471 | 0.000451 | 0.001662 |
| M1*: Structure of the core model 1; STMartin.INDEX=$St.MQoLS_{Total Score}$; CPT2: Dyskinetic Cerebral Palsy, CPT3: Ataxic Cerebral Palsy; CPT4: Unclassified Cerebral Palsy. | | | | | | |

**Figure S1: Scatterplot matrix between EQ-5D utility scores and domains of St. MQoL-S total scores**


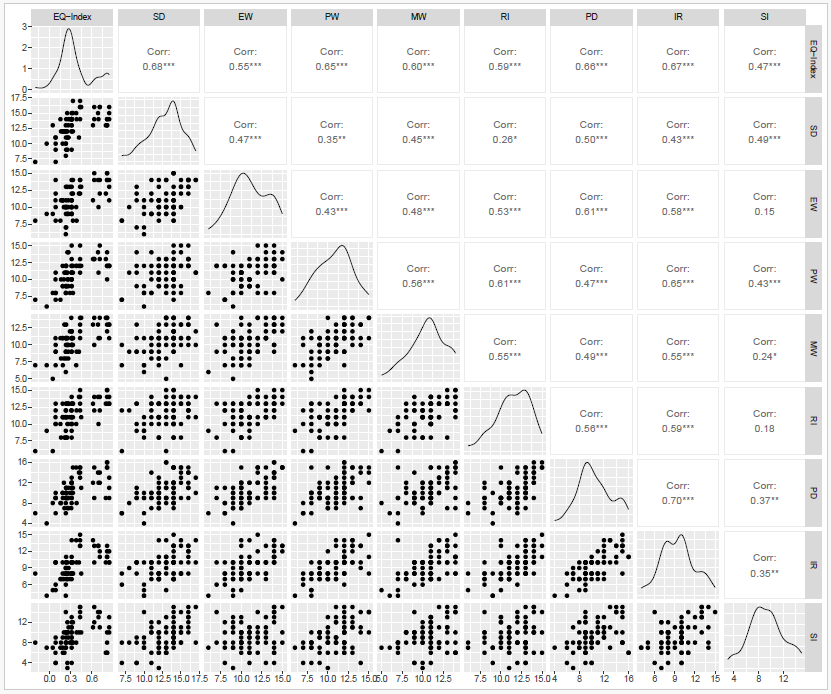


**Figure S2: Scatterplot between observed and predicted EQ-5D utility scores (Model 1)**


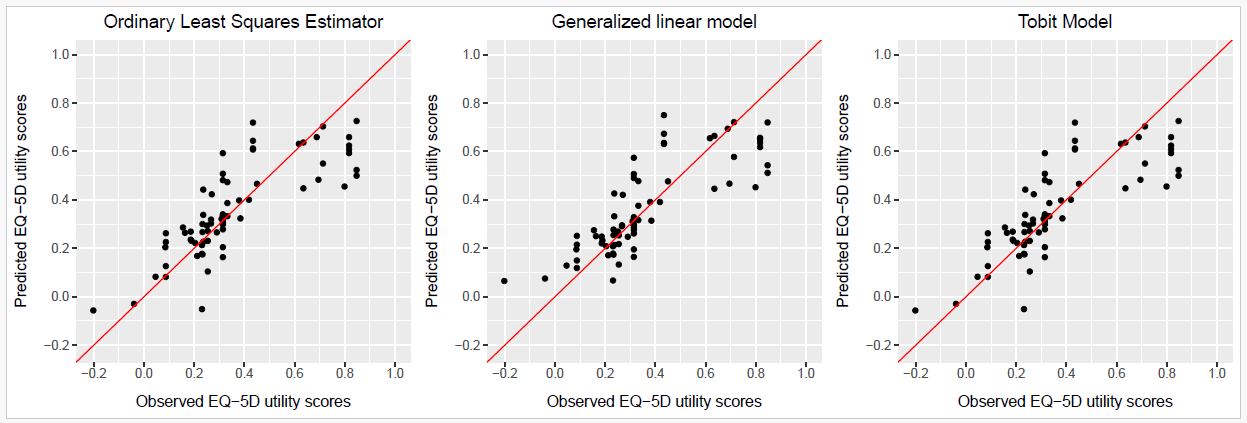


**Figure S3: Scatterplot between observed and predicted EQ-5D utility scores (Model 2)**


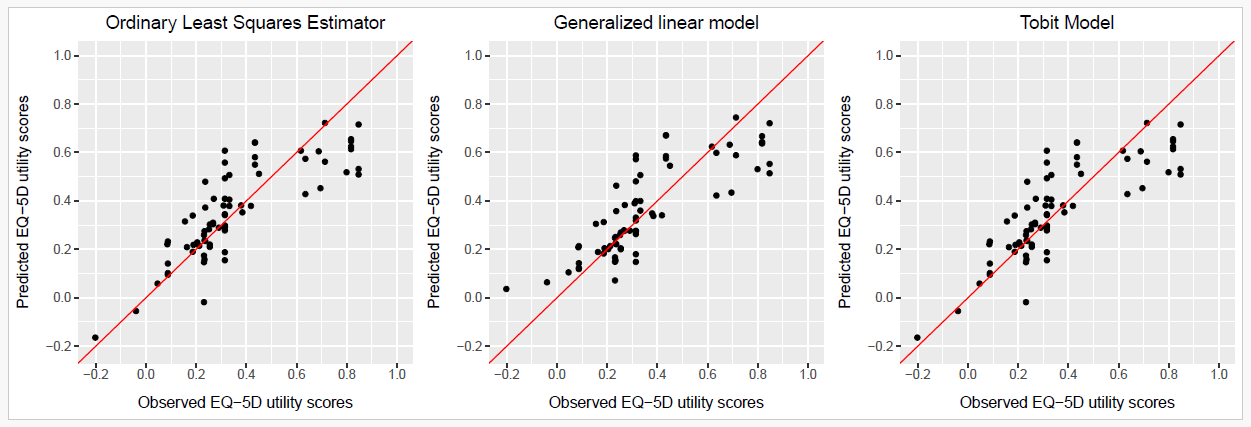

Supplement: Supplementary file 1 — Supplementary file1 (DOCX 298 KB) [file 10198_2025_1831_MOESM1_ESM.docx]
