# Supplementary material for: Estimating QALYs in adults with cerebral palsy: mapping the San Martin scale to the EQ-5D-5L for economic evaluation
Source: Eur J Health Econ. 2025 Sep 24;27(3):527–40. doi: 10.1007/s10198-025-01831-1 (PMC13190523; doi:10.1007/s10198-025-01831-1)
Supplement: Supplementary file 10 — Supplementary file10 (HTML 27 KB) [file 10198_2025_1831_MOESM10_ESM.html]

QALYs in adults with cerebral palsy: mapping from the San Martin Scale onto the EQ-5D-5L instrument


## Table of contents

- Data
- R code
  - Descriptive Analysis
  - Variable selection
  - Mapping model performance
  - Cross Validation
  - Examples of the mapping algorithm

# QALYs in adults with cerebral palsy: mapping from the San Martin Scale onto the EQ-5D-5L instrument

Author

Nova-Díaz, D.M., Adin, A., and Sánchez-Iriso, E.

Published

Jun/2025

This repository contains the original data and R code to reproduce the analyses presented in the paper entitled *“QALYs in adults with cerebral palsy: mapping from the San Martin Scale onto the EQ-5D-5L instrument”* (Nova-Díaz et al., 2025).

## Data

Data were collected from 72 participants in the Study of Adults Cerebral Palsy in Navarre, Spain (EPCANA), who completed both the St. MQoL-S and the EQ-5D-5L survey instruments simultaneously.

It can be loaded in R by using the command

```
library(xlsx)

Database <- read.xlsx("Data_EQ5DSTMF.xlsx", sheetIndex=1)
str(Database)
```

```
'data.frame':   72 obs. of  22 variables:
 $ ID                   : chr  "14" "62" "63" "44" ...
 $ SEX                  : chr  "1" "1" "0" "0" ...
 $ AGE                  : num  20 35 72 18 25 52 49 29 60 47 ...
 $ CITY                 : chr  "0" "1" "0" "0" ...
 $ CPT                  : chr  "1" "1" "1" "1" ...
 $ RLD                  : chr  "3" "3" "3" "3" ...
 $ TIME                 : num  14 33 27 6 6 29 40 26 19 9 ...
 $ INST                 : num  0.7 0.943 0.375 0.333 0.24 ...
 $ TOTAL.EQ             : num  23 21 20 21 19 19 19 19 19 18 ...
 $ EVA                  : num  55 55 55 58 70 60 58 60 55 55 ...
 $ EQ.INDEX             : num  -0.202 -0.04 0.046 0.085 0.087 ...
 $ STMartin.SD          : num  7 10 13 11 7 10 11 9 14 12 ...
 $ STMartin.EW          : num  8 9 9 11 8 10 14 12 8 11 ...
 $ STMartin.PW          : num  7 6 8 10 7 8 8 11 7 10 ...
 $ STMartin.MW          : num  7 7 5 11 10 7 9 9 11 8 ...
 $ STMartin.RI          : num  6 9 6 11 12 10 13 10 10 8 ...
 $ STMartin.PD          : num  6 4 8 9 6 9 10 9 8 8 ...
 $ STMartin.IR          : num  8 4 5 10 6 5 10 10 7 7 ...
 $ STMartin.SI          : num  8 7 7 6 4 8 4 9 7 8 ...
 $ STMartin.SUM         : num  57 56 61 79 60 67 79 79 72 72 ...
 $ STMartin.INDEX       : num  82 82 85 99 85 90 99 99 94 94 ...
 $ STMartin.INDEX.scaled: num  0.375 0.375 0.412 0.588 0.412 0.475 0.588 0.588 0.525 0.525 ...
```

The `Database` object contains the following variables:

- `ID`: character vector of patient identifiers
- `SEX`: gender of the patient (0=“Female”, 1=“Male”)
- `AGE`: age of the patient
- `CITY`: place of residence (0=“outside the city”, 1=“inside the city”)
- `CPT`: cerebral palsy type (0=“Spastic”, 1=“Dyskinetic”, 2=“Ataxic”, 3=“Unclassified”)
- `RLD`: recognized level of dependency (1=“Mild”, 2=“Moderate”, 3=“Severe”)
- `TIME`: time of affiliation with the institution (years)
- `INST`: degree of institutionalization (TIME/AGE)
- `TOTAL.EQ`: total dimension scores for EQ-5D-5L
- `EVA`: visual analog scale (0=Worst state / 100=Best state)
- `EQ.INDEX`: EQ-5D Index
- `STMartin.SD`: St. MQoL-S self-determination domain
- `STMartin.EW`: St. MQoL-S emotional well-being domain
- `STMartin.PW`: St. MQoL-S physical well-being domain
- `STMartin.MW`: St. MQoL-S material well-being domain
- `STMartin.RI`: St. MQoL-S rights domain
- `STMartin.PD`: St. MQoL-S personal development domain
- `STMartin.IR`: St. MQoL-S interpersonal relationships domain
- `STMartin.SI`: St. MQoL-S social inclusion domain
- `STMartin.SUM`: Sum of the St. MQoL-S domains
- `STMartin.INDEX`: St. MQoL-S total score
- `STMartin.INDEX.scaled`: St. MQoL-S total score scaled to [0,1] interval

## R code

The R code of this repository is organized according to the data analysis described in the paper.

### Descriptive Analysis

The script 1\_DescriptiveAnalysis.R performs a descriptive analysis of our dataset. Specifically, it generates the following figures and tables:

- **Table 1**: Characteristics of the sample (n=72)
- **Figure 2**: Distribution of EQ-5D utility score and St.MQoL-S total score
- **Figure 3**: Scatterplot between the EQ-5D utility scores and St.MQoL-S total scores
- **Table S2**: Spearman correlation coefficients between EQ-5D utility score and domains of St.MQoL-S total scores
- **Figure S1**: Scatterplot matrix between EQ-5D utility scores and domains of St.MQoL-S total scores

### Variable selection

In the scripts 2a\_VariableSelection\_OLS.R, 2b\_VariableSelection\_GLM.R and 2c\_VariableSelection\_Tobit.R we investigate which explanatory variables may serve as potential predictors of the EQ-5D utility score, using either the St. MQoL-S total score (core model 1) or its domain scores (core model 2) as the basis.

In all cases, ANOVA F-tests and stepwise regression are employed to evaluate alternative models and identify the most appropriate predictor variables.

### Mapping model performance

The script 3\_Mapping.R fits several direct mapping model candidates (using ordinary least squares, generalized linear model, and Tobit regression analysis) to estimate EQ-5D-5L utilities, with St. MQoL-S total and domain scores as explanatory variables.

It also generates the following figures and tables:

- **Table 2**: Goodness-of-fit results of model candidates
- **Table S3**: Estimated regression coefficients of model candidates
- **Figure S2**: Scatterplot between observed and predicted EQ-5D utility scores (Model 1)
- **Figure S3**: Scatterplot between observed and predicted EQ-5D utility scores (Model 2)
- **Figure 5**: Empirical cumulative density functions of observed and predicted EQ-5D utility scores (Model 1)

### Cross Validation

To ensure generalization for out-of-sample individuals, the script 4\_CrossValidation.R employs a repeated 5-fold cross-validation approach to select the mapping model with the best predictive performance.

Results are summarised in the figures and tables:

- **Table 3**: Goodness-of-fit using repeated 5-fold cross validation
- **Figure 4**: Goodness-of-fit using repeated 5-fold cross validation

### Examples of the mapping algorithm

Or best mapping algorithm (OLS - Model 1) can be described as

\[\begin{array}{rcl}
EQ.INDEX & = & -1.380 + 0.0157\*STMartin.INDEX + 0.0018\*AGE + 0.0016\*I(SEX=Male) + \\
& & 0.0775\*I(CPT=Dyskinetic) + 0.1658\*I(CPT=Ataxic) + 0.0842\*I(CPT=Unclassified)\\
\end{array}\]

where \(I(\cdot)\) denotes an indicator function.

After fitting the model, predictions of EQ-5D-5L utility values for an adult person with cerebral palsy can be obtained as follows:

```
## Fit the core model providing the best mapping algorithm: OLS - Model 1 ##
Model.final <- lm(EQ.INDEX ~ STMartin.INDEX + AGE + SEX + CPT, data=Database)
```

```
## EXAMPLE 1:
## - Prediction for a female (SEX="0") who has a St. MQoL-S total score of 106,
##   30 years old, and has a CP of the spastic type (CPT="1")
newdata <- data.frame(STMartin.INDEX=106, SEX="0", AGE=30, CPT="1")
newdata$EQ.INDEX <- predict(Model.final, newdata, interval="confidence")

print(newdata)
```

```
  STMartin.INDEX SEX AGE CPT EQ.INDEX.fit EQ.INDEX.lwr EQ.INDEX.upr
1            106   0  30   1    0.3357393    0.2738009    0.3976776
```

```
## EXAMPLE 2:
## - Prediction for a male (SEX="1") who has a St. MQoL-S total score of 100,
##   40 years old, and has a CP of the dyskinetic type (CPT="2")
newdata <- data.frame(STMartin.INDEX=100, SEX="1", AGE=40, CPT="2")
newdata$EQ.INDEX <- predict(Model.final, newdata, interval="confidence")

print(newdata)
```

```
  STMartin.INDEX SEX AGE CPT EQ.INDEX.fit EQ.INDEX.lwr EQ.INDEX.upr
1            100   1  40   2    0.3388744    0.1857931    0.4919557
```
